# Diabetes-Associated Dry Eye Syndrome in a New Humanized Transgenic Model of Type 1 Diabetes

**Published:** 2013-06-08

**Authors:** Shahnawaz Imam, Raya B. Elagin, Juan Carlos Jaume

**Affiliations:** Division of Endocrinology, Diabetes and Metabolism, Department of Medicine, School of Medicine and Veterans Affairs Medical Center, University of Wisconsin-Madison, Madison WI

## Abstract

**Purpose:**

Patients with Type 1 Diabetes (T1D) are at high risk of developing lacrimal gland dysfunction. We have developed a new model of human T1D using double-transgenic mice carrying HLA-DQ8 diabetes-susceptibility haplotype instead of mouse MHC-class II and expressing the human beta cell autoantigen Glutamic Acid Decarboxylase in pancreatic beta cells. We report here the development of dry eye syndrome (DES) after diabetes induction in our humanized transgenic model.

**Methods:**

Double-transgenic mice were immunized with DNA encoding human GAD65, either naked or in adenoviral vectors, to induce T1D. Mice monitored for development of diabetes developed lacrimal gland dysfunction.

**Results:**

Animals developed lacrimal gland disease (classically associated with diabetes in Non Obese Diabetic [NOD] mice and with T1D in humans) as they developed glucose intolerance and diabetes. Animals manifested obvious clinical signs of dry eye syndrome (DES), from corneal erosions to severe keratitis. Histological studies of peri-bulbar areas revealed lymphocytic infiltration of glandular structures. Indeed, infiltrative lesions were observed in lacrimal/Harderian glands within weeks following development of glucose intolerance. Lesions ranged from focal lymphocytic infiltration to complete acinar destruction. We observed a correlation between the severity of the pancreatic infiltration and the severity of the ocular disease.

**Conclusions:**

Our results demonstrate development of DES in association with antigen-specific insulitis and diabetes following immunization with clinically relevant human autoantigen concomitantly expressed in pancreatic beta cells of diabetes-susceptible mice. As in the NOD mouse model and as in human T1D, our animals developed diabetes-associated DES. This specific finding stresses the relevance of our model for studying these human diseases. We believe our model will facilitate studies to prevent/treat diabetes-associated DES as well as human diabetes.

## Introduction

Patients with diabetes are at significant risk of developing corneal lesions, such as superficial punctuate keratitis, recurrent corneal erosions, persistent epithelial defects, and microbial keratitis. These lesions are all due to lacrimal gland dysfunction [1]. Even proliferative diabetic retinopathy appears to have a more severe course in patients with diabetes and declined tear film function [2]. Whether or not lacrimal gland dysfunction is *metabolic* or *autoimmune* in origin is still controversial [3].

Proliferative and non-proliferative retinopathies are *metabolic* complications of diabetes. These *metabolic* complications are caused by microangiopathy, the severity of which in turn determines the development of retinopathy as well as nephropathy and neuropathy. However, a causative relationship between *metabolic* complications of diabetes and non-retinal ocular disease (i.e., lacrimal dysfunction) is not well established. On the contrary, evidence exists for the association between “tear film” dysfunction (characteristic of diabetes-associated dry eye disease) and *autoimmune* diabetes [4]. Studies have recently established that the prevalence of symptoms, signs, and definitive diagnosis of dry eye syndrome (DES) are higher, and basal tear secretion and tear film stability are lower, in children with *autoimmune* (type 1) diabetes mellitus (T1D), years before *metabolic* complications are even expected [4].

Animal models of autoimmune diabetes have provided the strongest evidence for a causative relationship between autoimmunity and diabetes-associated DES. Non-obese diabetic (NOD) mice, the classic model of human T1D, usually develop lacrimal gland dysfunction. Autoimmune lymphocytic infiltration of lacrimal glands accompanies lymphocytic infiltration of pancreatic islets in NOD mice [5]. A possible explanation for this association of pancreatic islets and lacrimal gland lymphocytic attacks has been based on the existence of cross-reactive antigens in both glands. Recently, a special group of CD4+ helper T cells was observed to be enriched in the inflamed pancreas and salivary glands of NOD mice as well as in the circulation of Sjögren’s syndrome patients [6].

We have developed a novel model of T1D using double-transgenic mice carrying HLA-DQ8 diabetes-susceptibility haplotype instead of mouse MHC-class II and expressing the human beta cell autoantigen Glutamic Acid Decarboxylase (GAD65) in pancreatic beta cells. Double-transgenic mice immunized with DNA encoding human GAD65 develop insulitis, glucose intolerance, and diabetes, while controls are insulitis-free and glucose-tolerant [7,8]. We report here the development of DES (classically associated with diabetes in NOD mice and proposed to be associated with T1D in humans) after diabetes induction in our humanized transgenic model.

## Methods

### Mice

HLA-DQA1*0301/DQB1*0302 (DQ8; murine MHC-class II molecule-deficient) and RIP7-hGAD65 line 1 transgenics in the BTBR background were used for all experiments [7,8]. All mice used in this study were >N6 crosses. All animal protocols were approved by the University of Wisconsin and the Veterans Affair animal research committees.

### Adenoviral constructs for immunization

Adenoviral constructs were made using the Gateway system (Invitrogen, Carlsbad, CA) as previously described [8]. All viruses used in this study were from the same preparation and were stored in aliquots at -80 °C. hGAD65 expression from pAD-CMVhGAD65 was confirmed by western blot. A pAD-CMV empty vector was used for control immunizations. Immunizations were performed two times at two-week intervals. Mice were intraperitoneally injected with 100 µl of PBS containing 10^11^ particles of pAD-CMVhGAD65.

### Histology, immunohistochemistry, and immunofluorescence

Pancreatic and lacrimal/Harderian gland sections were stained with hematoxylin/eosin for histological identification and localization of lymphocytic infiltration. Sections were then stained for markers of T cells (CD3) and B cell (CD20) lineage as well as macrophages (CD68 and MAC-1) using immunohistochemical techniques [8,9]. CD4 and CD8 markers were used to characterize helper versus cytotoxic T cell responses (CD4/CD8 ratio), mostly using immunofluorescence techniques and confocal microscopy, but also using histomorphometric microscopy [8,9]. The primary antibodies used were anti- CD3, CD4, and CD8 (1:100; BD PharMingen San Jose, CA).

IL-17 detection (anti-IL-17; 1:200; Santa Cruz Inc., Santa Cruz, CA), which identifies another subset of helper T cells known as Th-17 cells, was also performed. Finally, the presence of Foxp3 (anti-Foxp3; 1:200; Abcam, Cambridge, UK), a marker for T-regulatory cells, was investigated [8,9].

Lymphocytic infiltration was quantified by analyzing 15 microscopic fields. In the pancreas, it was expressed as percent of pancreatic islets infiltrated by lymphocytes. In lacrimal/Harderian glands, it was scored as severe when more than 50% of glandular tissue was affected by lymphocytic infiltration, and as mild when less than 50% was affected.

### Antibody measurement

Anti-GAD65 antibodies from mice serum samples were quantified with an ELISA assay according to manufacturer instructions (Kronus, Boise, ID). The ELISA results were validated with a parallel immunoprecipitation assay previously standardized in our laboratory [8]. Briefly, the immunoprecipitation method has been substituted entirely by the ELISA method. Control mouse serum samples showed less than 5 U/ml of anti-GAD65 antibody concentration. At least two independent experiments in duplicated wells were performed, and average values for each sample were obtained.

### Measurement of aqueous tear production

Tear production was measured with cotton threads (Zone-quick; Oasis, Glendora, CA). The threads were held with jeweler’s forceps and applied to the ocular surface in the lateral canthus for 60 s. Wetting of the thread was measured in millimeters, using the scale on the cotton thread under a light microscope [10].

### Statistical analysis

Two-tailed probability of chi-square distribution was used to compare results. P values were considered significant when <0.05.

## Results

We recently developed a new humanized animal model of autoimmune diabetes [7,8]. We conducted the current studies using ten transgenic and control mice. We followed an immunization protocol already shown to induce autoimmune diabetes in these mice [8]. Our animals developed eye problems as they developed glucose intolerance and diabetes. The glycemic level used to establish the diagnosis of diabetes was glucose >250 mg/dl in two consecutive days (as previously reported [8]). We observed a correlation between the severity of the pancreatic infiltration and severity of ocular disease (Table 1).

**Table 1:** DES and Severity of insulitis

Mouse #	Age (weeks)	Post- Immune		GAD65 Ab (units)	Islet Infiltration (%)*	Lacrimal/Harderian Infiltration†
		weeks	glucose‡			
1	26	23	262.5	265	**96**	OS **severe** OD mild
2	23	15	306	26.56	10	OS absent OD absent
3	27	18	254	16.27	**97**	OS **severe** OD mild
4	27	16	272	144	**82**	OS mild OD **severe**
5	26	23	258.5	295	76	OS mild OD absent
6	24	17	278	142	45	OS mild OD absent
7	27	16	296	28.67	26	OS mild OD absent
8	27	16	259	11.15	77	OS mild OD absent
9	22	12	271	163	**82**	OS **severe** OD **severe**
Ctr	32	25	168	5.53	0	OS absent OD absent

* Percent of pancreatic islets infiltrated by lymphocytes. † Intensity of infiltration was scored as severe when more than 50% of glandular tissue was affected by lymphocytic infiltration, mild when less than 50% was affected. ‡ Average of 2 consecutive glucose levels before euthanasia. **Bold** indicates association between severe infiltration of both lacrimal/Harderian glands and pancreatic islets. Animal numbers correspond to the same animals in Figure 3. Control is abbreviated as Ctr.

### Dry eye syndrome

**Figure 1:**
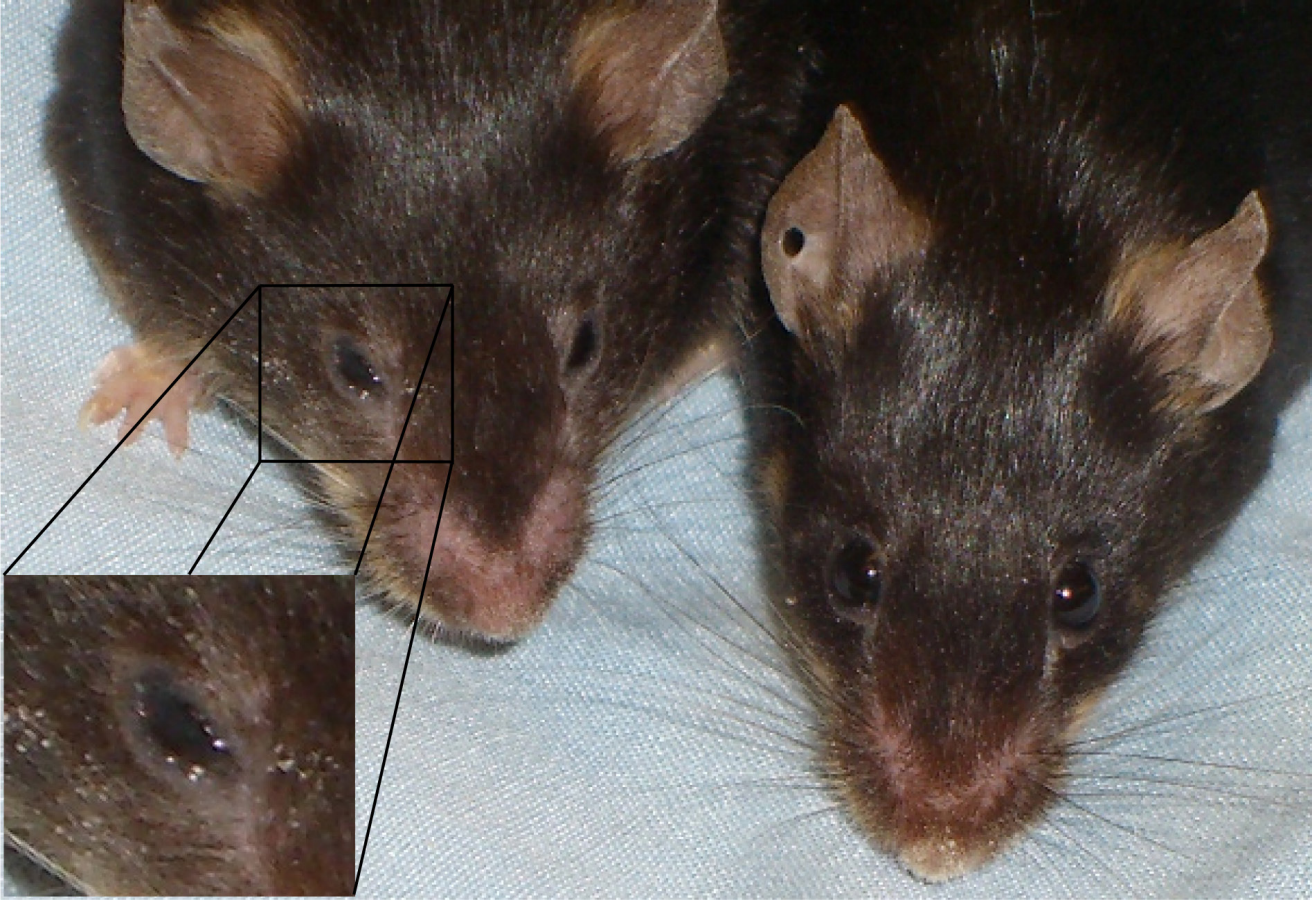
Diabetes-associated dry eye syndrome. Two transgenic mice (left immunized; right control) are shown. Close-up of affected animal shows early tear film dysfunction. Many of our animals developed eye manifestations soon after immunization. Our animals manifested obvious clinical signs of ocular disease, from corneal lesions to severe keratitis, before the onset of diabetes chronic metabolic disturbances.

**Figure 2:**
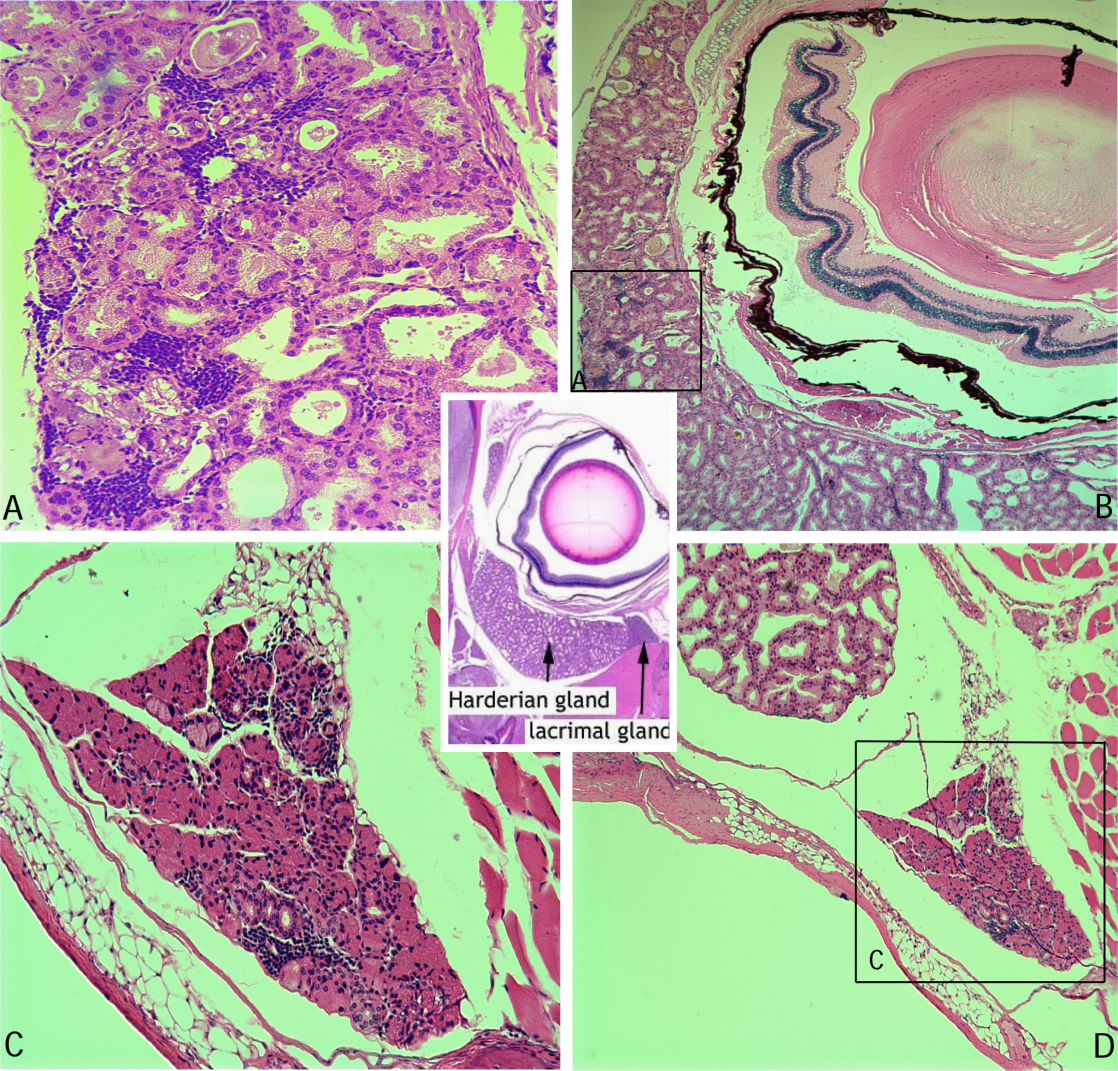
Lymphocytic infiltration of lacrimal and Harderian glands form transgenic mouse ten weeks post-immunization. Middle inset is a transverse image of the orbit for orientation for the location of the lacrimal and Harderian glands on normal mouse histology. **A**: Close-up of square also labeled A on **B**. **B**: Transverse section of the orbit, showing panoramic views of infiltration of the Harderian gland. **C**: Close-up of square also labeled C on **D**. **D**: Transverse section of the orbit, showing panoramic views of the infiltrated lacrimal gland. All specimens stained with hematoxylin/eosin.

Our animals developed dry eye signs several weeks after immunization for diabetes induction. From mild corneal erosions to severe keratitis, our animals manifested obvious clinical signs of ocular disease (Figure 1 is representative of early tear film dysfunction). DQ8/hGAD65 transgenic mice do not spontaneously develop diabetes or DES. However, upon immunization with a vector carrying hGAD65 coding DNA (induction), most animals develop insulitis, glucose intolerance, and diabetes [7,8]. Ten to twenty-four weeks post-immunization, animals were noted to develop clinical signs of tear film dysfunction (Figure 1). On further examination of histological specimens of the retro- and peri-bulbar areas, lymphocytic infiltration of glandular structures was revealed (Figure 2, early infiltrates). Indeed, severe histopathological lesions were observed in lacrimal and Harderian glands within weeks following development of glucose intolerance. Lymphocytic infiltration ranged from focal lesions to complete acinar destruction of the glands involved.

### Aqueous tear production defect

**Figure 3:**
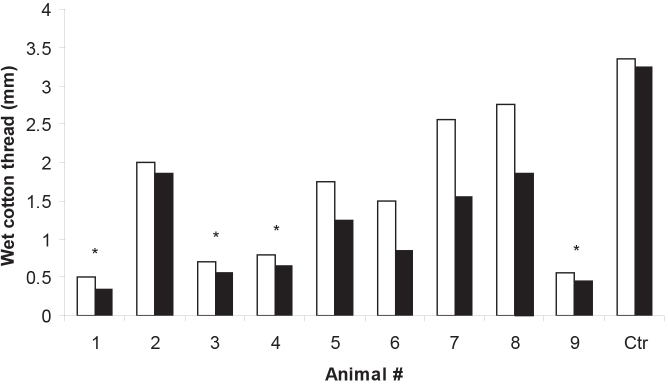
Modified Schrimer’s test. Tear production was measured with cotton threads. Columns represent thread wetting measured in millimeters on two occasions two weeks apart. Although all experimental animals had decreased tear production, four had significant differences (* p<0.05) when compared to control (Ctr.). Animal numbers correspond to the same animals in Table 1.

We performed two modified Schrimer’s tests using cotton threads [10] two weeks apart to assess for tear production defects ten weeks after triggering immunization with hGAD65. Most experimental animals demonstrated lower tear production when compared to controls (Figure 3). A statistically significant decrease in aqueous tear production for four animals (mean, 0.57±0.7 mm) in the experimental group, as compared with control animals (mean, 3.30±0.05 mm), was observed (Figure 3). Additionally, most experimental animals showed non-significant worsening of aqueous tear production within the two-week measurements. Notably, the mice with statistically significant defects in aqueous tear production also had the most severe lymphocytic infiltrates in lacrimal/Harderian glands (Table 1).

### Severity of insulitis correlates with DES

We observed a correlation between the severity of the ocular disease and the severity of the pancreatic islet lymphocytic infiltration (Table 1). The four animals most severely affected by DES carried also the most extensive pancreatic islet destruction (Table 1). GAD65 antibodies were present in the majority of the animals affected by the glandular lymphocytic infiltrate (lacrimal/Harderian and pancreatic islets). However, no correlation was found with the antibody titers and either lacrimal/Harderian or pancreatic islets lymphocytic infiltration/destruction.

### Immune characterization of lacrimal gland lesions

**Figure 4:**
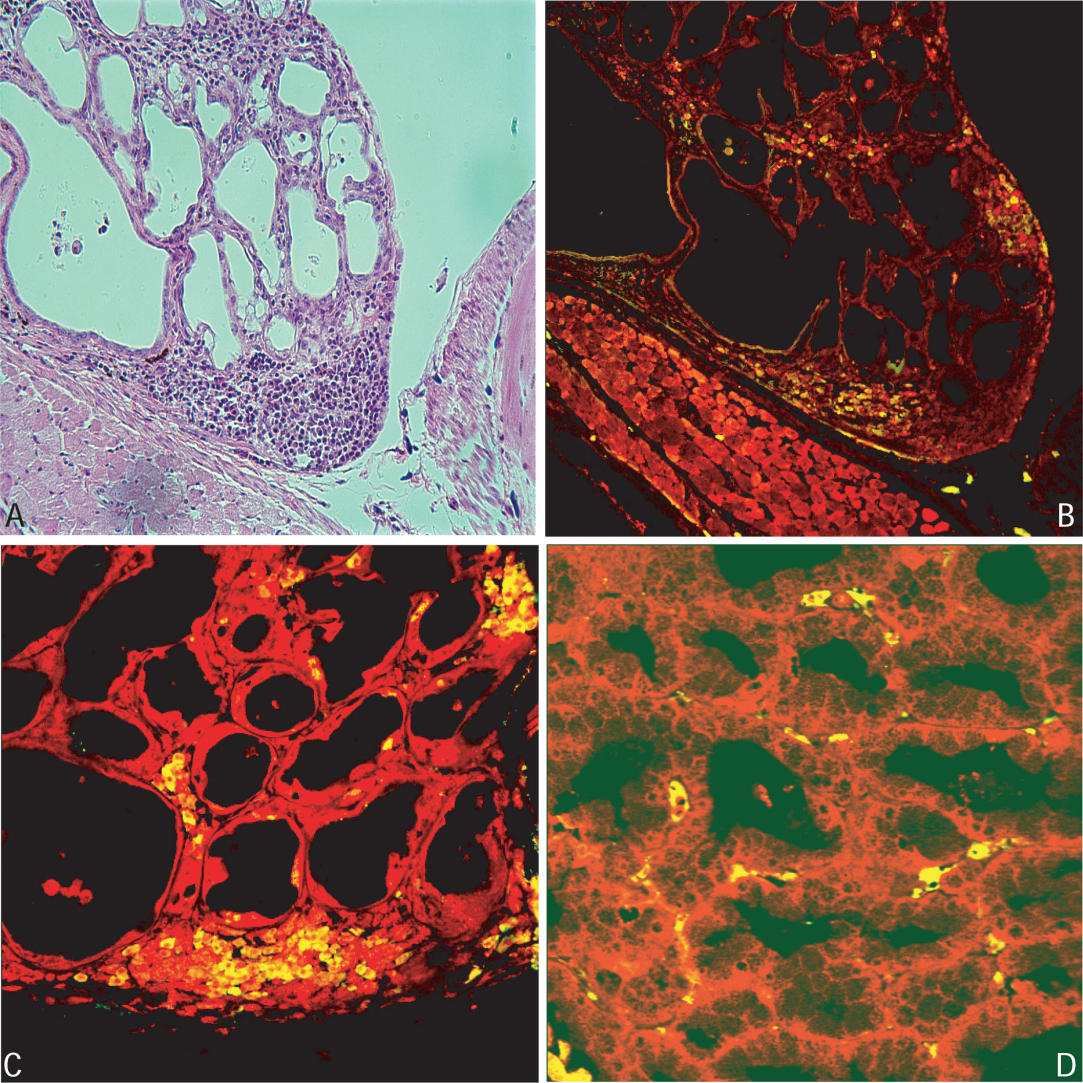
CD3 and CD4/CD8 staining of lacrimal gland of affected representative animal. Lymphocytic infiltration hematoxylin/eosin stained (**A**) was mostly represented by T cells. **B**: CD3 positive cells are shown in green (yellow on red nuclear staining background). CD4 positive T cells were abundant and concentrated in the area of infiltration (**C**), while CD8 positive cells were less abundant and sparser (**D**).

Lymphocytic infiltrates were characterized by immunohistochemistry and immunofluorescence. CD3-positive cells (T cells Figure 4B) were the dominant population in the infiltrates (Figure 4A). CD4+ T cells predominated over CD8+ in areas of heavy infiltrates (Figure 4C). CD8+ T cells were much less abundant and mainly present in inter-acinar spaces (Figure 4D).

**Figure 5:**
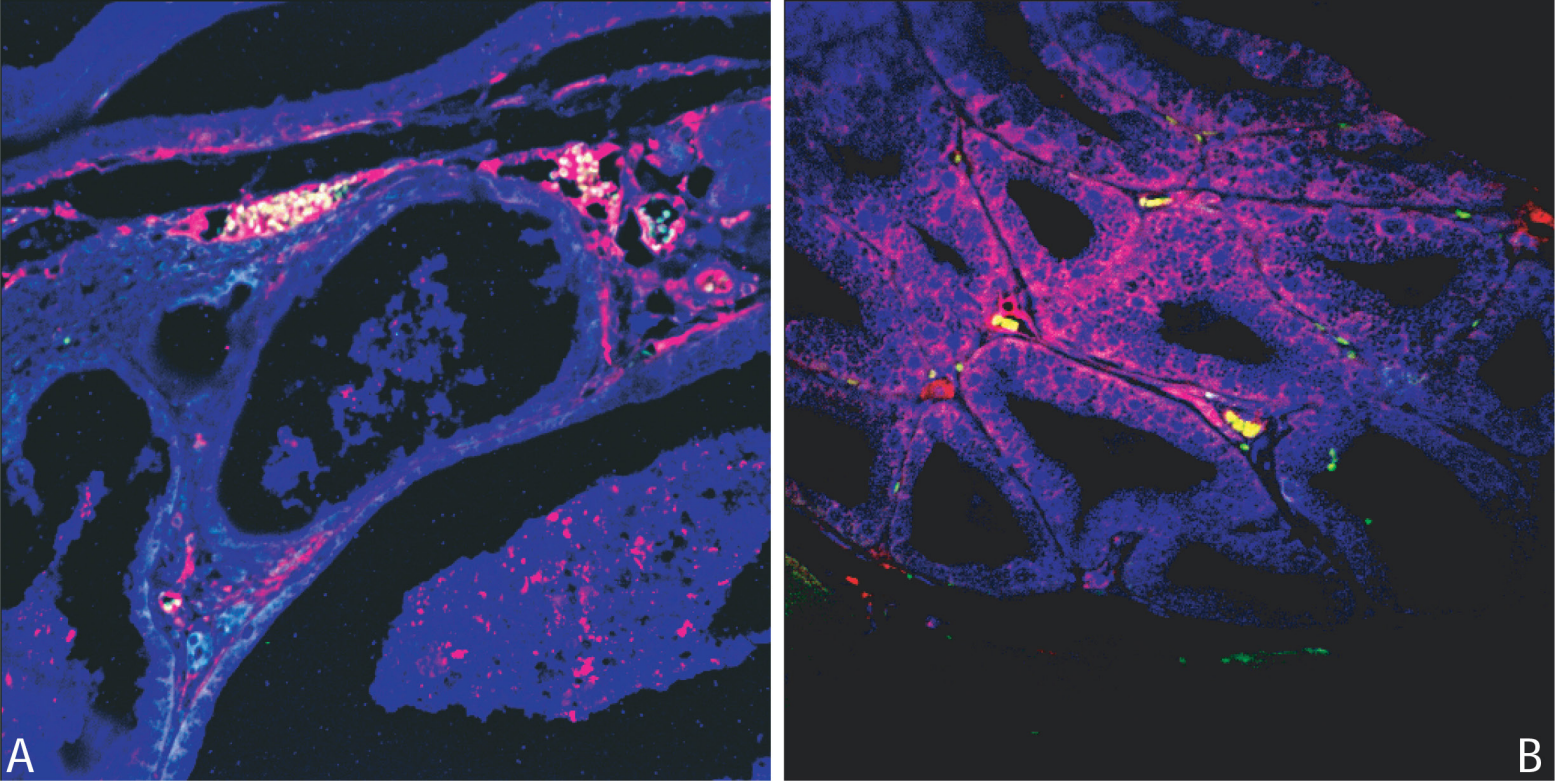
IL17/Foxp3 staining. Lacrimal glands of less-affected animals (**A**) revealed the presence of Foxp3 staining, while IL17 staining dominated in severely compromised glands (**B**).

We also studied the presence of IL17 and Foxp3 markers in trying to distinguish autoimmune versus immune-regulatory patterns. While the lacrimal/Harderian glands of severely affected animals were mainly positive for IL17 (Figure 5B), lacrimal/Harderian glands of animals with mild DES showed significant aggregates of Foxp3-positive cells (Figure 5A). Lacrimal/Harderian glands of control mice were free of infiltrates (not shown).

## Discussion

The association between diabetes and DES has been recognized for decades. Rudobielska et al. first suggested this association in 1973, when they published a case of Sjögren’s syndrome immediately followed by the development of diabetes in an eight-year-old girl [11]. Another case report followed in 1975, in which diabetes shortly preceded Sjögren’s [12]. In a larger 1990 study of 102 unselected T1D patients, 55% were affected by Sjögren’s [13]. Of those patients, 25% tested positive for antinuclear antibodies (HEp-2) and 32% were positive for anti-Ro antibodies, reinforcing the suspicion of an overall autoimmune rather than metabolic cause of DES [13]. Cases have been reported in which a common triggering event for both diabetes and DES was postulated [14]. Others have shown the concomitant occurrence of several autoimmune problems, highlighting the possibility that the association of autoimmune diabetes and DES was linked to a common autoimmune target [15,16]. The first large clinical study designed to compare the symptoms, signs, and results of objective tests for DES in T1D patients and controls was undertaken recently [4]. A total of 104 children with T1D and 104 age- and sex-matched controls were studied. A total of 15.4% of diabetic children complained of dry eye symptoms, versus 1.9% of controls (p=0.029). Dry eye signs were clinically detected in 7.7% of diabetic children, versus 0.96% of controls (p=0.034). Tear break-up time and Schrimer test of tear production were significantly lower (p=0.018, p=0.024, respectively) in the T1D group than in the control group. These results clearly demonstrated a statistically significant association of DES with T1D that preceded the metabolic complications of diabetes.

Lacrimal gland dysfunction has also been associated with autoimmune diabetes in animal models. Asamoto et al. observed in 1984 that lymphocytic infiltration of lacrimal glands accompanied lymphocytic infiltration of pancreatic islets in the NOD mouse model of autoimmune diabetes [17]. Furthermore, the NOD mouse has been proposed as a valid animal model for diabetes-associated DES or diabetes-associated Sjögren’s syndrome in the setting of autoimmune diabetes [18]. Unlike other mouse strains with reported infiltration of the lacrimal glands, such as MLR/lpr, MLR/+, and NZBxNZW F1 mice [19], gland dysfunction observed in NOD mice is concomitant with progressive cellular infiltration and tissue destruction of these organs. A possible explanation for the coexistence of pancreatic islets and lacrimal gland lymphocytic attacks has been based on the presence of candidate cross-reactive antigens in both pancreatic islets and lacrimal glands [20,21]. Unfortunately, the nature of the primary target antigen of the immune attack to the lacrimal glands in NOD mice is not known [22].

We have developed a diabetes mouse model, in which a known human autoantigen is presented to effector cells in the context of human diabetes-susceptibility genes [7,8]. After a triggering event, our transgenics develop insulitis and diabetes. A proportion of our animals also develop clinical DES in close association with their diabetes phenotype. Although the target antigen for the lacrimal gland attack is not known, GAD65 antigen is the target of the pancreatic islet attack. We first noticed dry eye signs when glucose tolerance tests confirmed incipient glucose homeostasis abnormalities. A modified Schrimer’s test confirmed the lack of proper tear secretion (tear film dysfunction). In the tested experimental group, most animals showed impaired tear secretion when compared to control mice, which was statistically significant for some. Mild corneal lesions evolved into severe keratitis in some animals. Upon development of diabetes, histological examination of the retro- and peri-bulbar specimens revealed lymphocytic infiltration of glandular structures that ranged from focal lesions to complete acinar destruction. As expected, the worse the tear secretion dysfunction, the more severe the lymphocytic infiltration in lacrimal/Harderian glands. The lymphocytic infiltration was predominantly of the T-cell type. CD4+ T helper cells seemed to be overrepresented compared to CD8+, and their distribution appeared somewhat different (focal for CD4+; sparse for CD8+). The proportion of cytotoxic (CD8+) T cells was not consistent with the tissue destruction in some cases. On further analysis of the T-helper (Th) cell type, we noticed subtle differences depending on the severity of the orbital gland infiltration. While lacrimal glands in severely affected animals showed the dominant presence of Th-17 cells (IL17 positive), mildly affected animals showed the presence of clusters of T regulatory cells (Foxp3 positive). Th-17 cells are thought to be good markers for autoimmune components of immune responses [23]. On the other hand, the presence of Foxp3 (a marker for T regulatory cells [24]) is believed to identify regulatory responses that when dominant, control autoimmunity. These differences may explain why we noticed more severe clinical features with more severe histology in animals lacking T regulatory cells.

We also noticed that more severe lacrimal gland dysfunction phenotypes accompanied more severe diabetes. Severity of lymphocytic infiltration of orbital glands correlated with severity of infiltration of pancreatic islets. This finding is in keeping with similar findings in the NOD model. In the NOD model, the severity of lymphocytic infiltration is directly correlated with the diabetes and DES phenotype [17]. This correlation points toward a common target between the orbital glands and pancreatic islets. In the NOD model, however, the target antigen is uncertain. In our model, GAD65 is the target antigen for the pancreatic islet attack. The immune response in our model is triggered by a peripheral immunization with the target antigen, which in turn homes to the pancreatic islets where the antigen is expressed. The transgenic expression of GAD65 outside the pancreatic beta cell has been studied, and non-measurable amounts of GAD65 have been detected anywhere else [25]. However, GAD expression around acinar cells in salivary glands has been demonstrated, and suggests that the GABAergic system plays a role in autonomic regulation of these and other glands [26]. Other cross-reactive antigens may also be implicated. Obvious and less obvious cross-reactive candidate antigens that have been investigated include islet cell autoantigens (ICA) 69, prominin-1 (CD133), and insulin. ICA69 is a self-antigen expressed in brain, pancreas, salivary, and lacrimal glands. An inactivate genomic ICA69 locus in the NOD strain generated NOD-congenic mice that were deficient in ICA69; in addition, it prevented lacrimal gland disease. Immunotherapy with a high-affinity mimicry peptide targeting ICA69-specific T-cells reduced established Sjögren’s syndrome in wild-type NOD mice [20]. Prominin-1 (CD133) is expressed by various stem and progenitor cells originating from diverse sources. In addition to stem cells, its mouse ortholog is expressed in a broad range of adult epithelial cells. Two independent laboratories have demonstrated expression in secretory serous cells, mucous cells, and intercalated ducts of lacrimal glands, as well as all duct-lining cells of the pancreas [21,27]. Insulin has been shown to be secreted in the tear film; its mRNA is expressed in the lacrimal gland and its receptor in tissues of the ocular surface [28,29]. Furthermore, insulin production in the lacrimal glands, showed by: (i) the presence of insulin and C-peptide, (ii) glucose- and carbachol-induced insulin secretion ex-vivo and (iii) biochemical and histological characteristics of diabetic lacrimal glands that would support this possibility, have been demonstrated [30]. Indeed, after inducing diabetes with a streptozotocin injection, diabetic male Wistar rats had rising insulin levels in the tear film after glucose stimulation. Ex vivo static secretion assays demonstrated that higher glucose and carbachol significantly increased mean insulin levels from lacrimal glands. Insulin and C-peptide were expressed in diabetic rats’ lacrimal glands, as determined by RIA. All of these candidate cross-reactive antigens still need to be further investigated to find the cause of the association between diabetes and DES.

Our results demonstrate development of DES in association with antigen-specific insulitis and diabetes following immunization with clinically relevant human autoantigen, concomitantly expressed in mouse beta cells. As in the NOD mouse model and as in human T1D, our animals developed diabetes-associated DES. This specific finding stresses the relevance of our model for studying the human disease. We believe our model will facilitate studies to prevent human diabetes and other associated syndromes, such as DES.
